# Lipid droplet binding thalidomide analogs activate endoplasmic reticulum stress and suppress hepatocellular carcinoma in a chemically induced transgenic mouse model

**DOI:** 10.1186/1476-511X-12-175

**Published:** 2013-11-22

**Authors:** Lajos I Nagy, Eszter Molnár, Iván Kanizsai, Ramóna Madácsi, Béla Ózsvári, Liliána Z Fehér, Gabriella Fábián, Annamária Marton, Csaba Vizler, Ferhan Ayaydin, Klára Kitajka, László Hackler, Lajos Mátés, Ferenc Deák, Ibolya Kiss, László G Puskás

**Affiliations:** 1 AVIDIN Ltd., Szeged, Hungary; 2 AVICOR Ltd., Szeged, Hungary; 3 Institute of Biochemistry, Biological Research Centre of the Hungarian Academy of Sciences, Szeged, Hungary; 4 Institute of Plant Biology, Biological Research Centre of the Hungarian Academy of Sciences, Szeged, Hungary; 5 Institute of Genetics, Biological Research Centre of the Hungarian Academy of Sciences, H-6726, Temesvári krt. 62, Szeged, Hungary

**Keywords:** Hepatocellular carcinoma, Lipid droplet, Heat-shock proteins, Protein disulfide isomerase, Reactive oxigen species

## Abstract

**Background:**

Hepatocellular carcinoma (HCC) is the most frequent and aggressive primary tumor of the liver and it has limited treatment options.

**Results:**

In this study, we report the *in vitro* and *in vivo* effects of two novel amino-trifluoro-phtalimide analogs, Ac-915 and Ac-2010. Both compounds bind lipid droplets and endoplasmic reticulum membrane, and interact with several proteins with chaperone functions (HSP60, HSP70, HSP90, and protein disulfide isomerase) as determined by affinity chromatography and resonant waveguide optical biosensor technology. Both compounds inhibited protein disulfide isomerase activity and induced cell death of different HCC cells at sub or low micromolar ranges detected by classical biochemical end-point assay as well as with real-time label-free measurements. Besides cell proliferation inhibiton, analogs also inhibited cell migration even at 250 nM. Relative biodistribution of the analogs was analysed in native tissue sections of different organs after administration of drugs, and by using fluorescent confocal microscopy based on the inherent blue fluorescence of the compounds. The analogs mainly accumulated in the liver. The effects of Ac-915 and Ac-2010 were also demonstrated on the advanced stages of hepatocarcinogenesis in a transgenic mouse model of N-nitrosodiethylamine (DEN)-induced HCC. Significantly less tumor development was found in the livers of the Ac-915- or Ac-2010-treated groups compared with control mice, characterized by less liver tumor incidence, fewer tumors and smaller tumor size.

**Conclusion:**

These results imply that these amino-trifluoro-phthalimide analogs could serve potent clinical candidates against HCC alone or in combination with dietary polyunsaturated fatty acids.

## Background

Hepatocellular carcinoma (HCC) is the fifth most common cancer of men, while the eighth most frequent cancer of women worldwide, and the second leading cause of cancer death [1,2]. The majority of HCC cases are associated with chronic hepatitis or cirrhosis induced by persistent infection with hepatitis B or hepatitis C virus [3]. Despite advances in different chemotherapies which are often associated with toxic side effects, liver cancer has limited treatment options. More effective therapeutic agents with fewer side effects are in the focus of current research.

Novel thalidomide analogs, Ac-915 and Ac-2010 (4-amino-substituted 2,6-diisopropylphenyl- -5,6,7-trifluoroisoindole-1,3-diones) were synthesized from starting tetrafluoro thalidomide based on our previously published synthetic approach [4]. Both molecules possessed a strong blue fluorescence, like the previously synthesized ones [4], and in the present study their intracellular and tissue distribution were detected based on their fluorescent characteristics. Here, we demonstrated that Ac-915 and Ac-2010, novel amino-trifluoro-phtalimide analogs with novel substitutions also interfere with lipid droplets and the endoplasmic reticulum (ER), and induce intracellular reactive oxygen species (ROS) at lower concentrations than the previously described compounds. The novel compounds described here, specifically interact with lipid droplet-associated proteins, protein disulfide isomerase (PDI) and heat-shock proteins (HSPs) that are involved in chaperone functions. The upregulation of HSPs, as observed in various cancers, including liver cancer suggests that they might be involved in carcinogenesis [5-7]. Knockdown of PDI activity can cause accumulation of misfolded proteins in the ER, activation of apoptotic signaling, and induction of caspase-dependent apoptosis in breast cancer cells [8]. Here we investigated the possible PDI inhibition of the novel analogs by using enzymatic assays.

Lipid droplets (LDs) are one of the main intracellular targets of amino-trifluoro-phtalimide analogs. It was shown that LD accumulation occurs *in vivo* in prenecrotic cancer tissues, therefore LDs can serve as *in vivo* markers of cancer [9]. Imbalance in lipid homeostasis can finally lead to membrane disruption and activation of lipoapoptosis [10]. As LDs are formed in the ER we were able to show that specific LD-binding drugs could interfere with LD homeostasis and ER-membrane integrity and could trigger apoptosis through ER stress [4].

Tumors, including hepatocellular carcinoma are more sensitive to ER stress and reactive oxygen species (ROS)-inducing natural compounds, such as polyunsaturated fatty acids (PUFAs) and celestrol among others [4,11] than normal cells as their stress response is continuously engaged due to their chronic stress situation, thereby leading to activation of pro-apoptotic signals and finally tumor cell death [12].

Loss of lipid droplets in hepatic stellate cells is one of the first events observed in the development of liver disease leading to HCC, mostly due to the dramatic drop in cellular retinyl ester content [13]. Surprisingly, in study Blaner and co-workers showed that Lrat KO mice, which lacks the sole enzyme responsible for hepatic retinyl ester synthesis, showed significantly less liver tumor development compared with wild-type mice, as characterized by less liver tumor incidence and smaller tumor size [14].

On the contrary, the liver of matrilin-2 KO (*Matn2*^−/−^) mice contained macroscopic tumors of both larger number and size than the wild-type liver after diethylnitrosoamine (DEN) treatment. DEN is widely used as a carcinogen in experimental animal models. Upon intraperitoneal administration into weaning mice at 2 weeks after birth, hepatic tumors are formed 8 month later [15]. Since DEN itself does not exert carcinogenicity, it needs to be bioactivated by cytochrome P450 enzymes in the liver, resulting in DNA-adducts that form through an alkylation mechanism locally, which induce the formation of putative preneoplastic lesions [16].

Owing to the increased number and size of the DEN-induced liver tumors in the transgenic *Matn2*^−/−^ mice, we used this *in vivo* model to assess the efficacy of our novel amino-trifluoro-phtalimide analogs. The worthwhile *in vivo* efficacy data presented here anticipate the completion of pre-clinical studies and initiate a clinical study on evaluating the effects of Ac-915, or the more potent Ac-2010 analog in humans with high risk for liver carcinoma.

## Material and methods

### Cell culture studies

Hep3B and HepG2 cells were grown in mixture of Dulbecco’s Modified Eagle Medium (D-MEM) (high glucose) (Gibco BRL, Carlsbad, CA, USA) and Nutrient Mixture F-12 Ham (Sigma, St. Louis, MO, USA) containing penicillin (50 IU/ml)–streptomycin (50 mg/ml) and 10% fetal bovine serum. For cytotoxicity assays, cells were seeded at a density of 10.000 cells per well into 96-well cell culture plates and maintained in a humidified atmosphere of 95% air and 5% CO_2_ for 12 h, then treated with different concentrations of Ac-2010 or Ac-915. MTS (3-(4,5-dimethylthiazol-2-yl)-5-(3-carboxymethoxy-phenyl)-2-(4-sulfophenyl)-2H-tetrazolium) assay was applied to drug treated and control (0.2% DMSO) cells with CellTiter 96® AQueous Assay (Promega, Madison, WI) according to the manufacturer’s protocol.

### Intracellular localization

HepG2 cells were cultured in glass bottom culture dishes (MatTek Corporation, Ashland, MA). ER was labeled in live cells with ER-Tracker Green (Invitrogen, Carlsbad, CA) following the manufacturer’s protocol. After staining the solution was replaced by fresh Hank’s Balanced Salt Solution with calcium and magnesium containing 5 μM Ac-915 or Ac-2010 and 5 min later the cells were visualized using an Olympus Fluoview FV1000 confocal laser scanning microscope equipped with 20x (N.A 0.75) and 40x oil (N.A 1.3) objectives. We applied a 543 nm laser for detection of the ER-Tracker Green and 405 nm laser for detection of Ac compounds [17].

### Tissue distribution

Mice were injected i.v. with Ac-915 or Ac-2010 (20 mg/kg) and after different time points tissues were dissected, washed in PBS, embedded in Tissue-Tek® O.C.T™ medium. After fast-freezing, the samples were stored at −20°C. Tissue sections (20 μm) were prepared by using a Leica CM1950 cryostat at −20°C. From each tissue (liver, brain, kidney, muscle, heart) three independent images were recorded as above. Images were analyzed with the Olympus Fluoview 1.6 Ver Viewer software.

### Affinity chromatography and protein identification

Ac-201 compound, a previously described trifluoro-amino-phtalimide analog [4] was covalently attached to activated controlled pore glass resins by using the AviLink™ technology (Avicor, Szeged, Hungary, http://www.avicorbiotech.com) [18]. After blocking and washing the columns, whole cell lysate from 6×10^7^ RVH cells was applied onto the column with 1 ml resin in PBS containing protease inhibitors. After washing the columns with 20 ml each of 0.2 M and 0.5 M NaCl in PBS, proteins were eluted with 1% SDS in PBS and applied to polyacrylamide gel-electrophoresis. Specific protein bands were cut out and stored at 4°C until further processing for mass spectrometry analysis. Samples were processed for mass spectrometry by using the UCSF in-gel digestion protocol (http://donatello.ucsf.edu/ingel.html). LC-MS analysis was performed by using the Agilent 1100 LC-MS/MS system with Zorbax 300SB-C18, 5 μm, 5mmx0,3 mm and Zorbax 300SB-C18, 3.5 μm, 150x0,075 mm columns). MS-method: Scan: m/z 300–1600, MS2 Scan: m/z 100–1800.

### Waveguide optical biosensor assay

Protein immobilization was accomplished by applying 10 μL of 50 μg/mL human PDI (Sino Biological Inc., Beijing, China) or 50 μg/mL human HSP70 (Sino Biological) or 100 μg/mL human HSP90 (Sino Biological) in 20 mM sodium acetate pH 5.5 into a preactivated 384-well biochemical plate (Perkin Elmer) and incubating overnight at 4°C. The microplates were subsequently washed three times with assay buffer (PBS, pH 7.4/0.005% Tween 20/3% DMSO) and, after 25 minutes thermal equilibration, a baseline reading was taken in the Enspire Multimode Plate Reader (Perkin Elmer). Finally, 15 μL assay buffer was added including different concentrations of Ac-915 or Ac-2010. The Enspire response is measured as a shift in reflected wavelength and is expressed in picometers (pm). The affinity (K_D_) was calculated using a curve fitting method.

### Protein disulfide isomerase assay

The assay is based on the measurement of the catalytic reduction of insulin as described by Lundstrom and Holmgren [19]. In this assay, PDI facilitates the reduction of insulin in the presence of DTT. The reduced insulin chains aggregate, and the turbidity is monitored spectrophotometrically at 650 nm. The assay was performed in a 96-well plate format and a volume of 30 μl in the presence of 1 mM DTT, 1 μg PDI (Sino Biological), 0.14 mM bovine pancreas insulin (Sigma), and 0.2 mM EDTA in 100 mM potassium phosphate, pH 7.0. The progress of the reaction was monitored on a 96-well plate reader (Model 680 Microplate Reader, Bio-Rad Laboratories) at 650 nm for 95 minutes at 25°C. Ac-915, Ac-2010 or DMSO control was added prior to the addition of enzyme at the concentrations indicated. The nonenzymatic reduction of insulin by DTT was recorded in a control well without PDI. IC_50_ values of the compounds were calculated using nonlinear regression analysis.

### Real-time cellular analysis with the xCELLigence System

Cytotoxicity and cell migration was monitored with the real-time cell electronic sensing, xCELLigence System (RT-CES, Acea-Roche) [20,21]. Cytotoxicity effects of Ac-2010 and Ac-915 compounds were tested on two different hepatocellular carcinoma cell lines (HepG2 and Hep3B). The RT-CES 96-well E-plate was coated with gelatin and then washed twice with PBS. Growth media (respective to cell types) were then gently dispensed into each well of the 96-well E-plate for background readings by the RT-CES system prior to addition of cell suspension at a density of 6000 cells/well. Devices containing the cell suspension were kept at 37° in a CO_2_ incubator for 8 h prior to treatment with different concentrations of Ac-2010 and Ac-915. Cell growth was monitored for 48 h by measurements of electrical impedance every 5 min.

For migration assay, 160 μl of media containing 10% FBS was added to the lower chambers of CIM-plate 16. Wells of the upper chamber sealed at the bottom with a micro-pore-containing polycarbonate were filled with Hep3B cell suspension (20,000 cells/well) in 100 μl serum-free medium. Cell migration to lower chamber was monitored and expressed as cell index value. Continuous recording of impedance in cells of both systems was reflected by cell index value.

### ROS determinations

ROS generation was determined by the increase in DCFDA (2',7'-dichlorodihydrofluorescein diacetate, Sigma) fluorescence after drug stimulation. HepG2 cells were washed, resuspended in 1% bovine serum albumin in Hanks buffered saline solution (BSA-HBSS) at 10^6^ cells/mL and maintained at 37°C for analysis. Cells were treated with the indicated drugs (Ac-915, Ac-2010). DCFDA was added 60 min prior to harvest, at 2 μM final concentration and intracellular ROS production was measured by using a fluorescence activated cell sorter (Beckton Dickinson) with 20,000 events/test. All the other parameters and calculations were as described before [4].

### Glutathione measurements

HepG2 cells (20000 cells per well in a 96-well plate) were incubated with Ac-915 in 50 μl PBS for 2 hours in a humidified atmosphere of 95% air and 5% CO_2_. 50 μl aliquots of prepared 2X GSH-GloTM Reagent (GSH-GloTM Glutathione assay; Promega, Madison, WI) were added to the wells and incubation continued at room temperature for 30 minutes. 100 μl of reconstituted Luciferin Detection Reagent (Promega) was added to each well and cells were incubated for 15 minutes further. Negative controls and blank reactions were also prepared. The amount of light produced was detected by luminometer.

### Animal maintenance and treatments

All mice were fed a commercial diet and water ad libitum and were housed in an animal facility under a 12 h light/dark cycle at constant temperature and humidity. For all of our studies, we employed male Matn2-deficient mice (*Matn2*^−/−^) congenic in the 129/Sv genetic background [22].

For studies of liver tumor development, 15-day-old mice were treated with a single dose of DEN (Sigma-Aldrich, Budapest, Hungary) dissolved in saline at a dose of 25 mg/kg body weight by i.p. injection. 4 months after DEN injection mice were treated with either Ac-915 for 3 months (n = 15) or with Ac-2010 for 1 month (n = 10). Mice were killed 8 months after DEN administration for determination of tumor occurrence (number of tumor nodules) and liver mass index (liver weight/body weight × 100). Treatments were conducted by i.p. injection of Ac-915 at a dose of 10 mg/kg body weight three times a week or Ac-2010 at a dose of 4 mg/kg body weight three times a week.

### Animal ethic

The animal experiments were performed according to Institutional and National Animal Experimentation and Ethics Guidelines in possession of an ethical clearance (XVI/03047-2/2008).

## Results

### *In vitro* effect on cell proliferation and migration

**Figure 1:**
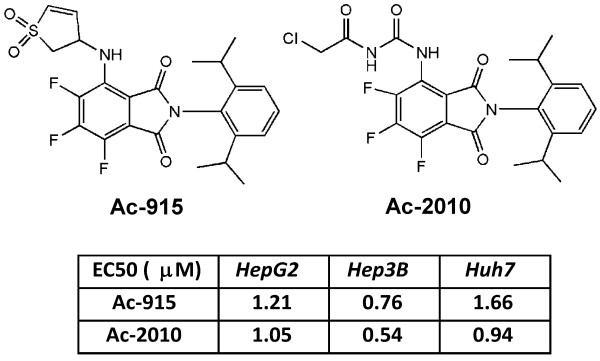
**Chemical structure of trifluoro-amino-phthalimides: Ac-915 and Ac-2010 and their EC**_**50 **_**values in different liver cancer cell lines.**

Two novel amino-trifluoro-phtalimide analogs synthesized by Avidin Ltd. Ac-915 (2-[2,6-bis(propane-2-yl)phenyl]-4-{[(3R)-1,1-dioxo-2,3-dihydro-thiophene-3-yl]amino}-5,6,7-trifluoro-2,3-dihydro-1H-isoindole-1,3-dione) and Ac-2010 (2-[2,6-bis(propane-2-yl)phenyl]- 5,6,7-trifluoro-1,3-dioxo-2,3-dihydro-1H-isoindole-4-yl}-3-(2-chloroacetyl)urea) showed superior cytotoxic activity in cancer cells and therefore were selected to the present study. Their cytotoxic effects on human hepatocellular carcinoma cell lines (namely: HepG2, Hep3B and Huh7) were measured by using the MTS assay. EC_50_ values for 48 h exposure were summarized next to their chemical structures in Figure 1. Both Ac-915 and Ac-2010 induced cell death of liver cancer cells at sub- or low micromolar ranges.

**Figure 2:**
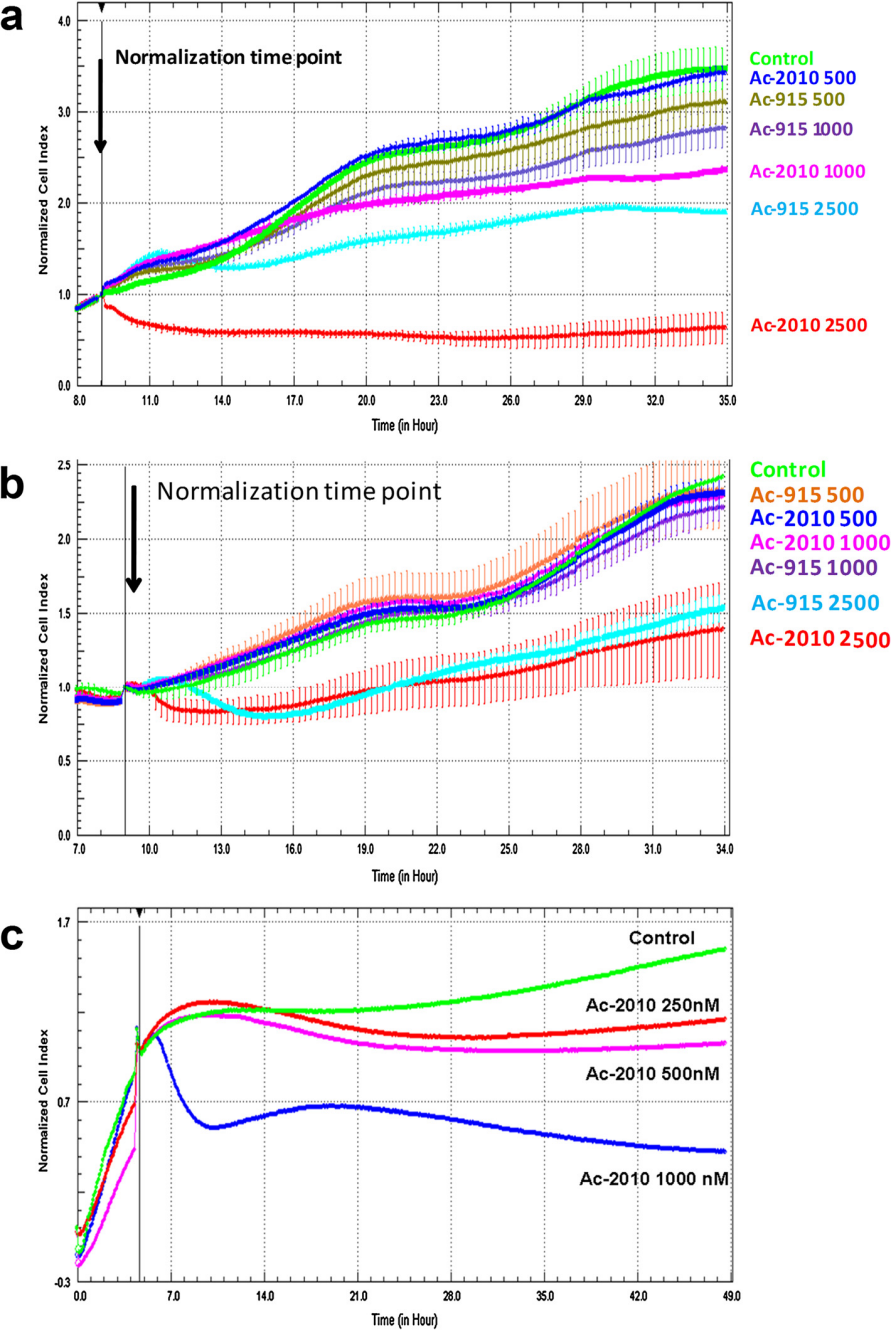
**Real-time analysis of cytotoxicity of Ac-915 and Ac-2010 against (a) Huh7 and (b) HepG2 cells.** Arrow shows the starting point of treatment of the cells. Each cell index value was normalized to this starting point. **(c)** Real-time analysis of the inhibitory effects on HepG2 cell migration. Average cell index values were calculated from four biological replicas. Standard deviation of each value is shown in each curve. (Numbers after compound codes are concentrations in nM).

Cytotoxic effects of Ac-915 and Ac-2010 compounds were also tested by the real-time cell electronic sensing, xCELLigence System (RT-CES) on two different hepatocellular carcinoma cell lines (Huh7, HepG2). This technology is based on proprietary microelectronic cell sensor arrays that are integrated in the bottom of the microtiter plates (96-well E-plate). When cells are cultured in a well, impedance is measured between sensor electrodes and the attached cells that act as insulators, which is converted into cell index number [20,21]. As shown in Figure 2a both analogs exerted micromolar cytotoxic effects on both liver cancer cell lines used. These results are in good correlation with data obtained by using the biochemical assay.

To determine whether our novel compounds have only effects on cell proliferation or they inhibit cell migration, the same technology was used. Cell migration was followed in real-time by using the RTCA DP xCELLigence System (Acea-Roche). This is a novel cell migration and invasion assay system that uses the Boyden Chamber principle but the bottom chamber has a micro-pore-containing polycarbonate membrane, which contains microelectronic sensor arrays on its bottom surface. Migration of cells is detected when cells go through these electrodes, which changes impedance, and will increase cell index. As seen in Figure 2b Ac-2010 inhibited migration even at 250 nM, where no cytotoxicity could be detected in case of HepG2 cells. At higher concentration the drug completely inhibited cell migration 2 h after administration.

### Mechanism of action

To gain insight into the association of the new analogs with their physiological targets, we identified target proteins in total cell lysates of human tumor cells by affinity chromatography. In order to determine the interacting proteins of trifluoro-amino-phtalimides the AviLink™ technology (Avicor Ltd.) was applied that enabled us to prepare affinity resins. After washing and elution of the columns, the eluates were analyzed on polyacrylamide gels. Gel pieces corresponding to specific protein targets were cut out and submitted to mass spectrometry-based protein identification. The results are summarized in Table 1. Among the hits we found several proteins that were previously shown to be lipid droplet-associated (such as fatty acid synthase, SEC22B, HSP60, HSP70, HSP90, calnexin, β-tubulin, RAB7, RAB10, RAB11, and protein disulfide-isomerase A6 (PDI)). Other proteins (Reticulon-3, SEC22B, and Neutral alpha-glucosidase AB) were previously shown to be localized to ER and Golgi.

**Table 1:** Trifluoro-phtalimide-associated proteins from human cancer cells

**Entry name**	**Function**	**Subcellular location**
14-3-3 protein epsilon	Signal transduction	Cytoplasm, lipid droplet
Aldehyde dehydrogenase, mitochondrial	Alcohol metabolism	Mitochondrium, lipid droplet
Calcyclin-binding protein	Proteosomal degradation of proteins	Nucleus, cytoplasm
Calnexin	MHCI antigen-binding	Lipid droplet
Dimethylaniline monooxygenase	Oxidation of drugs, pesticides, xenobiotics	Microsome
Hsp 60	Chaperone	Mitochondrium, lipid droplet
Hsp 70 kDa protein 5 (GRP 78)	Chaperone	ER, lipid droplet
Hsp 90-alpha	Chaperone	Cytoplasm, lipid droplet
Hsp 90-beta	Chaperone	Cytoplasm, lipid droplet
Leucine-rich repeat-containing protein 59	Regulate nuclear import	Microsome
Long-chain-fatty-acid-CoA ligase 3	Lipid biosynthesis, fatty acid degradation	Mitochondrium, peroxisome, microsome, lipid droplet
Membrane-associated progesterone receptor 1	Receptor for progesterone	Microsome
Neutral alpha-glucosidase AB precursor	Maturation of glycoproteins	ER, Golgi
Phosphate carrier protein, mitochondrial	Phosphate transport	Mitochondrium
Prenylcysteine oxidase	Degradation of prenylated proteins	Lysosome
Protein disulfide-isomerase A6	Chaperone	ER, lipid droplet
Protein FAM10A5	Suppression of tumorigenicity	Cytoplasm
Ras-related protein Rab-7	Vesicular trafficking	Endosome, lysosome, phagosome, lipid droplet
Ras-related protein Rab-10	Vesicular trafficking, neurotransmitter release	Cell membrane, lipid droplet
Ras-related protein Rab-11B	Vesicular trafficking	Cell membrane, lipid droplet
Reticulon-3	Membrane trafficking	ER, Golgi
Sorting nexin-2	Intracellular trafficking	Endosome
T-complex protein 1 subunit zeta	Chaperone	Cytoplasm
Twinfilin-1	Motile, morphological processes, endocytosis	Cytoplasm
Tubulin beta-2 chain	Cytoskeleton, vesicular transport	Cytoplasm, lipid droplet
UDP-glucuronosyltransferase 1-6	Elimination of toxic xenobiotics	Microsome
Vesicle-trafficking protein SEC22b	Vesicular trafficking	ER, Golgi, lipid droplet

In Table 1 the possible function of the protein targets can be also seen. The protein target functions can be classified in three major classes: 1) vesicular and membrane trafficking, 2) chaperone functions, and 3) elimination of toxic compounds.

Direct biomolecular interactions were determined between human recombinant and purified HSP70, HSP90, PDI proteins and Ac-915 and Ac-2010 with resonant waveguide optical biosensor technology in a biochemical binding assay [23]. The technology is based on a microplate with resonant waveguide optical biosensors integrated into each well and a high throughput screening-compatible optical reader (Enspire system, Perkin Elmer). The biochemical interaction between the small molecule and the protein was measured as a shift in reflected wavelength and is expressed in picometers (pm). The binding of both analogs to each of the protein was specific and saturable with the dose response (data not shown). The affinity (K_D_) values were calculated as follows for Ac-915: HSP70 K_D_: 14 μM, HSP90 K_D_: 11.5 μM and PDI K_D_: ≈10.5 μM; for Ac-2010: HSP70 K_D_: ≈16 μM, HSP90 K_D_: ≈16 μM and PDI K_D_: ≈6 μM.

The upregulation of HSPs as observed in various cancers, including liver cancer suggests that they might be involved in carcinogenesis. In particular, the enhancement of carcinogenesis via the overexpression of HSP60, HSP70 and HSP90 has been previously implicated in animal models [9] and in clinical samples [5-7,24]. Because of the stressful cancerous microenvironment, tumor cells strived to increase the expression of chaperone proteins for cytoprotective function and to enhance tumor growth and metastasis, therefore inhibition of their chaperone function is a logical option for cancer treatment.

**Figure 3:**
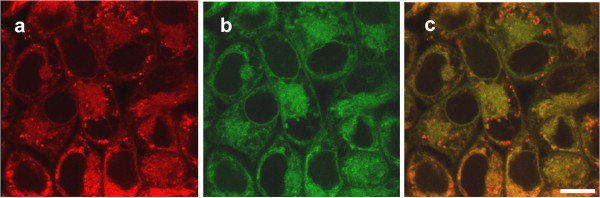
**Intracellular localization of Ac-2010. (a)** HepG2 human hepatocellular carcinoma cells were double-stained with the blue fluorescent Ac-2010 (10 μM) pseudocolored as red, and **(b)** endoplasmic reticulum-specific dye (ER-TrackerTM Green). **(c)** Orange color derives from colocalization of ER-TrackerTM and Ac-2010. Red spots correspond to lipid droplets as ER-tracker does not stain oil bodies, while Ac-2010 does. Scalebar is 10 μm.

As amino-trifluoro-phtalimide thalidomide analogs possess strong inherent blue fluorescence, intracellular localization can be easily performed on live cells. The subcellular localization of the new thalidomide analogs Ac-915 and Ac-2010 was determined by fluorescent microscopy in human liver cancer cells. Previously we determined that amino-trifluoro-phtalimides stain lipid droplets and some of their derivatives were co-localized to ER [4,17]. In order to assess the localization of the two novel compounds, we incubated HepG2 human HCC cells in culture medium containing Ac-915 or Ac-2010 and performed fluorescent microscopy. Both compounds showed ER-specific localization as presented for Ac-2010 in Figure 3a-c. Ac-2010 pseudocolored red gives yellow signals upon colocalization with ER-specific green signals. Red dots inside the cell correspond to lipid droplets that ER tracker does not stain.

Intracellular localization studies were in good concordance with our target identification results, where most of our hits were localized to ER or lipid droplets.

Based on the affinity target identification and co-localization results, PDI was selected to study whether our novel amino-trifluoro-phtalimide analogs influence its enzymatic activity. PDI is a multifunctional 57-kDa oxidoreductase of the thioredoxin superfamily that catalyzes the formation, cleavage and rearrangement of disulfide bonds and, by acting as a molecular chaperone, it facilitates protein folding [25]. PDI is expressed mainly in the ER of eukaryotic cells, where it predominantly forms disulfide bonds in nascent secretory proteins. It catalyzes the rearrangement of incorrect disulfide bonds through isomerase activity, thus supporting proper protein folding mainly during cellular stress, but during normal cellular conditions as well [26]. Loss of PDI activity can cause accumulation of misfolded proteins in the ER, i.e. ER stress and subsequently induce cell death. Hashida et al. presented that PDI-knockdown activated apoptotic signaling, resulting mitochondrial cytochrome c release, activation of different caspases, and finally it induces caspase-dependent apoptosis in MCF7 cells [8].

As anticipated both Ac-915 and Ac-2010 inhibited the activity of PDI with about 15 μM and 9 μM IC_50_ values, respectively. These values are in good correlation with the K_D_ values determined by direct biochemical interaction measurements using waveguide optical biosensors. The activity of PDI was based on the measurement of the catalytic reduction of insulin as described by Lundstrom and Holmgren [19]. In this assay both analogs inhibited the PDI-induced reduction of insulin in the presence of dithiothreitol. The reduced insulin chains aggregated slower in the presence of the analogs, when compared to untreated samples due to the slower insulin reduction (data not shown). Although IC_50_ values are higher than the effective concentration inducing ROS, GSH depletion and cytotoxicity, we assume that both Ac-915 and Ac-2010 are localized in the ER and their local, subcellular concentrations could be much higher. Therefore appropriate inhibition of PDI can be achieved at relative lower concentrations applied to cells.

**Figure 4:**
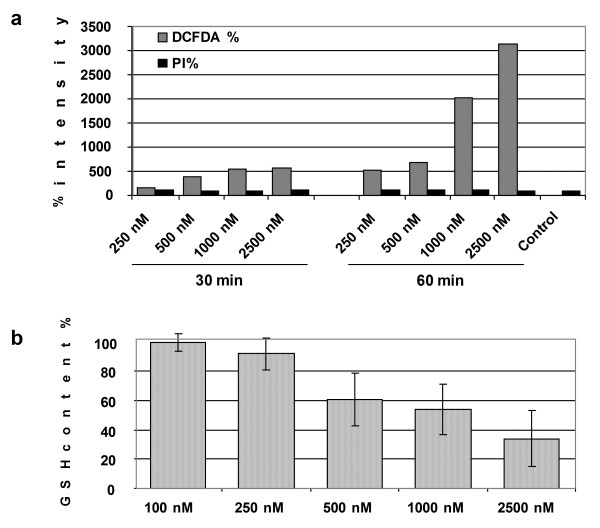
**Intracellular glutathione (GSH) concentrations in Ac-915 treated Hep3B cells. (a)** GSH content is shown relative to values obtained with the untreated control (%). **(b)** ROS formation (DCFDA %) and cell death (PI %) after treatment of Hep3B cells with Ac-915 at different concentrations 30 min and 60 min after treatment.

Toxic compounds and numerous anticancer agents interfere with chaperone and ER functions leading to cellular stress which is manifested by elevated reactive oxygen species (ROS) and dramatic decrease in the anti-oxidant, glutathione (GSH) level. To investigate whether Ac-915 and Ac-2010 exert a pro-oxidative effect as determined earlier for other amino-trifluoro-phthalimides [4] and “redox-reactive” thalidomide analogs [27], we measured the presence of ROS in human hepatocellular carcinoma cells (Hep3B) Figure 4a. To reveal the correlation of depletion of glutathione and ROS production of the analogs we determined the intracellular concentrations of glutathione. To determine whether Ac-915 and Ac-2010 affect intracellular GSH levels Hep3B cells were treated with compounds and GSH levels were recorded. According to our expectations, by inducing oxidative stress both compounds also depleted intracellular GSH levels (for Ac-915 GSH levels, see Figure 4b.

There is increasing evidence that ER stress plays a crucial role in the regulation of apoptosis in cancer and normal cells [28]. ER stress leads to the production of misfolded proteins, and as a consequence to alleviate ER stress different chaperones and folding enzymes are up-regulated. During this response, several pro-apoptotic signals are activated and, depending on the extent of the ER stress, cells undergo apoptosis [29]. Our results further support our hypothesis that our novel amino-trifluoro-phtalimide analogs interact with the ER and proteins associated with it, they induce ER stress, ROS production, decreased glutathione level and finally cell death.

### Tissue distribution

**Figure 5:**
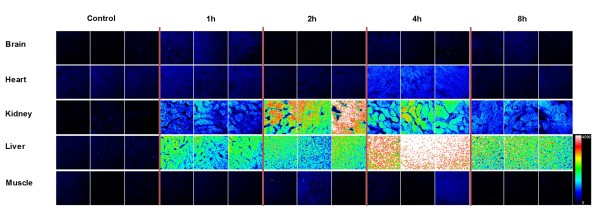
**Tissue distribution of Ac-915.** Tissue distribution of Ac-915 as determined by using fluorescent confocal microscopy on native tissue samples prepared after i.v. drug administration. Dimensions of each image tile is 125×125 μm.

To determine the tissue distribution of trifluor-amino-phtalimides, compounds were administered by i.v. injection into mice, the animals were killed at different time points and different organs were dissected. Native microscope sections were prepared and analyzed under fluorescent microscope. Because our analogs have strong inherent blue fluorescence, relative tissue distribution can be determined by using fluorescent microscopy with 405 nm laser with DAPI configuration. As all tissues possess inherent blue fluorescence at different intensities, fluorescent signals of untreated control animal samples were compared with those of treated samples. Results are shown in Figure 5. There was only a slight fluorescence increase 1 h after administration of Ac-915 in the brain, which implies that these analogs cannot penetrate to the brain. There was minor fluorescence increase in the heart, but only 4 h after administration. The target organs of Ac-915 seemed to be the kidney and preferentially, the liver. Ac-915 exhibited strong fluorescent signals in the liver with a peak at 4 h. Similarly, 2 and 4 h after administration strong signals could be recorder in the kidney.

From these results we concluded that the novel trifluor-amino-phtalimides indeed accumulate in the liver, the target organ of our cancer study.

### *In vivo* effects on DEN-induced hepatocarcinoma model

Based on the increased number and size of the DEN-induced liver tumors in the transgenic Matn2^−/−^ mice compared to the wild-type mice, the transgenic model was used to assess the *in vivo* effects of our potent amino-trifluor-phthalimide analogs.

**Figure 6:**
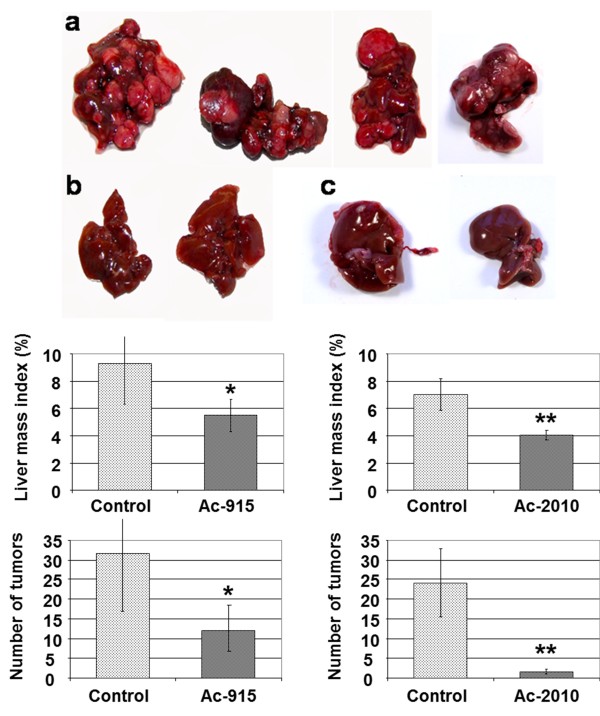
***In vivo *effects of Ac-915 and Ac-2010 on DEN-induced carcinogenesis. (a)** Representative liver samples from DEN-induced and un-treated, Ac-915-treated **(b)**, and Ac-2010-treated animals **(c)**. Liver mass index and number of tumors in samples were also determined.

For studies of liver tumor development, 15-day-old mice were treated with a single dose of DEN as described previously [15]. Mice in the Ac-915 treated group (n = 15) were treated 4 months after DEN treatment for an additional 3 months. Treatments were conducted by i.p. injection of Ac-915 at a dose of 10 mg/kg. Mice were killed 8 months after DEN administration and the number and size of tumors and liver mass index were determined. Representative photographs of the livers of DEN-induced non-treated controls and Ac-915-treated mice are shown in Figure 6a and b, respectively. Upon assessment of liver tumors, we found significantly less tumor development in the livers of the treated mice compared with that of control mice, as evaluated by less liver tumor incidence, fewer tumors and smaller tumor size which was also represented by the liver mass index.

To investigate whether the other analog, Ac-2010 may exhibit similar activity the same experiment was replicated, but instead of applying the drug for 3 months Ac-2010 was administered only for 1 month (n = 10). Liver samples were analyzed at the same time point, 8 months after DEN administration. Because of higher acute toxicity and better solubility (data not shown), Ac-2010 was injected at a lower dose (4 mg/kg) three times a week. Even with this protocol we obtained better result, than with Ac-915. Significantly (p < 0.01) less liver tumor incidence, fewer tumors and lower liver mass index was obtained compared to the untreated control group Figure 6c.

Drug administration was started 4 months after DEN administration, when the early stages of carcinogenesis ended and pre-neoplastic foci already appeared. Our data imply that Ac-915 or Ac-2010 treated mice experience less cell proliferation and cancer progression at later stages of liver cancer development. These findings may open a novel chemotherapeutic intervention for patients with the cancerous stage and these analogs may be useful in preventing HCC development.

## Discussion

HCC is the most frequent and aggressive primary tumor of the liver and it has limited treatment options. The present study demonstrated a potent cell death-inducing effect of two novel amino-trifluoro-phtalimide analogs, Ac-915 and Ac-2010. Amino-trifluoro-phthalimides, which bind lipid droplets, induce intracellular ROS formation and ER-stress [4]. Both Ac-915 and Ac-2010 compounds induced cell death of liver cancer cells at sub or low micromolar ranges detected by classical biochemical end-point assay as well as with real-time measurements. Besides cell proliferation inhibition, analogs exert cell migration inhibition even at 250 nM. Cytotoxic effects of the novel analogs were mediated by affecting chaperone functions, induction of oxidative stress and depletion of intracellular GSH. The novel amino-trifluoro-phthalimides interacted with several proteins that localized into lipid droplets and ER. Among their candidate protein targets are the different heat-shock proteins (HSP60, HSP70, and HSP90) and protein disulfide isomerase (PDI). Direct biomolecular interactions between human HSP70, HSP90, PDI proteins and Ac-915 and Ac-2010 were confirmed with resonant waveguide optical biosensors. The activity of PDI was inhibited by both Ac-915 and Ac-2010 at micromolar concentrations.

Relative biodistribution of the analogs was analyzed in using native tissue sections of different organs after administration of drugs, and fluorescent confocal microscopy based on the inherent blue fluorescence of the compounds. The target organs of the analogs were the liver and the kidney. No, or minimal penetration could be detected into the brain, the muscle or the heart.

We used the Matn2^−/−^ mice and DEN treatment for induction of tumors in the liver. Mice were treated either with Ac-915 (10 mg/kg) for 3 months, or Ac-2010 (4 mg/kg) for 1 months, following 4 months of DEN treatment. Liver tumor assessment was conducted 8 months after DEN administration. Significantly less tumor development was found in the livers of the Ac-915- or Ac-2010-treated groups compared with those of control mice, and were characterized by less liver tumor incidence, fewer tumors and smaller tumor size. These results indicate that treatment with either Ac-915 or with Ac-2010 affected tumor progression at later stages, which implies that these amino-trifluoro-phthalimide analogs alone or in combination with agents or natural compounds that influence ER-stress could serve potent clinical candidates against HCC.

## Competing interests

The authors declare that they have no competing interests.

## Authors’ contributions

LIN: carried out microelectronic sensing and waveguide optical biosensor studies. EM: carried out affinity chromatography and protein identification studies. IK: participated in the design and coordination of chemical synthesis and carried out synthesis of Ac compounds. RM: carried out synthesis of Ac compounds. BÓ: coordinated microelectronic sensing studies. LZF: carried out molecule screenings. GF: carried out molecule screenings. AM: carried out animal maintenance and treatments. CV: coordinate the treatment of animals. FA: carried out microscopic studies. KK: participated in the design of the study and performed the statistical analysis. LH: participated in the design of the study and performed the statistical analysis. LM: participated in the design of the study and performed the statistical analysis. FD: participated in the design of the study and drafted the manuscript. IK: participated in the design of the study and drafted the manuscript. LGP: participated in the design of the study and drafted the manuscript. All authors read and approved the final manuscript.
